# Biomimetic Surface Engineering Strategies for Enhanced Osseointegration and Peri-Implant Bone Regeneration: A Systematic Review

**DOI:** 10.3390/biomimetics11070460

**Published:** 2026-07-02

**Authors:** Fatma Karacaoğlu, Zülal Deniz Güner, Merter Güçlü, Elif Didem Özer, Nilsun Bağış, Kaan Orhan

**Affiliations:** 1 Department of Periodontology, Faculty of Dentistry, Ankara University, Yenimahalle, Ankara 06560, Turkey; fboke@ankara.edu.tr (F.K.); edozer@ankara.edu.tr (E.D.Ö.); nbagis@ankara.edu.tr (N.B.); 2 Department of Periodontology, Faculty of Dentistry, Bozok University, Yozgat 66100, Turkey; merter.guclu@bozok.edu.tr; 3 Department of Dento Maxillofacial Radiology, Faculty of Dentistry, Ankara University, Yenimahalle, Ankara 06560, Turkey; call53@yahoo.com; 4 Ankara University Medical Design Application and Research Center (MEDITAM), Ankara University, Ankara 06010, Turkey; 5 Department of Oral Radiology, School and Hospital of Stomatology, Cheeloo College of Medicine, Shandong University, Jinan 250012, China

**Keywords:** dental implant, biomimetics, bio-inspired surfaces, osseointegration, implant surface engineering, surface modifications

## Abstract

Objective: This systematic review aimed to evaluate the effects of biomimetic surface engineering strategies applied to dental implants on osseointegration and peri-implant bone regeneration compared with conventional implant surfaces. Materials and Methods: A comprehensive literature search was conducted in the Web of Science, PubMed, and Scopus databases in accordance with the PRISMA guidelines, covering the period from January 2021 to January 2026. A total of 12 studies, including in vivo animal experiments and in vitro investigations, that met the inclusion criteria were analyzed. Risk of bias assessment was performed using the SYR-CLE tool and the ARRIVE guidelines. Results: Biomimetic strategies, including laser texturing, sulfonation, bioactive coatings, and growth factor/peptide functionalization (e.g., BMP-2, FGF-2, and PRF), significantly increased bone–implant contact (BIC), new bone volume (BV/TV), and biomechanical stability (pullout strength and reverse torque) compared to conventional surfaces. These surfaces enhance fixation under conditions of low bone density, such as osteoporosis, and improve infection resistance through antibacterial activity. In addition, these modifications enhance cellular adhesion, osteogenic differentiation, angiogenesis, and immune modulation. Conclusions: Current experimental evidence suggests that biomimetic implant surface engineering transforms dental implants from passive biomaterials into multifunctional bioactive interfaces capable of simultaneously regulating osteogenesis, immune response, angiogenesis, and antibacterial activity. Although promising outcomes have been demonstrated in preclinical studies, standardized long-term human clinical studies are still required to validate translational potential and long-term clinical efficacy.

## 1. Introduction

Dental implants have become a widely accepted and preferred solution for replacing missing teeth, providing both functional and esthetic benefits [1]. Although the success rate of dental implants has been reported to be approximately 95% in the literature, failures—particularly those associated with insufficient osseointegration—remain clinically significant [1,2,3]. Bone quality, biocompatibility of the implant material, surgical procedure, and surface characteristics play critical roles in the osseointegration of dental implants. After implant placement, the rate of bone response largely depends on the quality of the implant surface properties [4]. Surface characteristics, such as composition, topography, roughness, and surface energy, influence the mechanical stability of the bone–implant interface and osseointegration at the histological level [5,6]. The quality of the implant surface may increase surface roughness or create microvoids that facilitate the integration of macromolecules with bone, thereby determining the biological response of bone tissue to implantation in the oral cavity. Various dental implant surface modifications have been developed to improve the interaction between the implant surface and the surrounding biological environment to enhance osseointegration [7]. Surface modifications are generally classified into physical, chemical, and biochemical alterations.

Physical modifications applied to improve the clinical success and stability of dental implants are categorized into macro-, micro-, and nanoscale levels [8]. Macro-level modifications (such as tapered implant shapes, wider diameters, and V-shaped thread designs) optimize the mechanical interlocking between the bone and implant, thereby enhancing primary stability during placement and improving stress distribution within the bone [9,10]. Micro-level modifications, including machining, sandblasting, and sandblasted large-grit acid-etched (SLA) techniques, increase the implant surface area at the micrometer scale and create a conductive scaffold for osteogenic cells, thereby promoting bone growth and secondary integration [10,11,12]. However, increased micro-level surface roughness may elevate the risk of bacterial adhesion and biofilm formation [13]. To address this challenge and further enhance cellular integration, nano-level modifications, such as laser ablation and nanocomposite coatings, have been developed. These modifications improve surface wettability and protein adsorption, thereby promoting osteoblastic differentiation and osseointegration, significantly reducing bacterial adhesion, and preventing infection [11,12,14].

Recent advances in surface engineering technologies have enabled the development of next-generation biomimetic dental implants with enhanced biological performances. In addition to conventional sandblasting and acid-etching procedures, several advanced fabrication and coating technologies such as discrete crystalline deposition (DCD), and treatments involving fluoride, hydroxyapatite, and calcium chloride [8] are increasingly being used to create bioactive and multifunctional implant interfaces. DCD-modified implant surfaces have been reported to reduce bacterial adhesion [15] and increase bone–implant contact (BIC) owing to enhanced nanotopography [16].

In addition to the aforementioned approaches, recent advancements in surface engineering have introduced sophisticated chemical modification techniques such as anodization, electrophoretic deposition, and chemical vapor deposition (CVD). Anodization enables the formation of highly ordered TiO_2_ nanotube arrays that significantly enhance osteoblast adhesion, proliferation, differentiation, and surface wettability [17,18,19]. Similarly, electrophoretic deposition facilitates the homogeneous coating of bioactive materials, such as hydroxyapatite, graphene, and composite nanomaterials, thereby improving surface bioactivity and interfacial bonding [20,21]. Chemical vapor deposition provides precise control over the coating thickness and composition, contributing to the improved mechanical stability, corrosion resistance, and long-term durability of implant surfaces [22]. In addition, these techniques, UV photofunctionalization and plasma treatments, increase surface hydrophilicity, thereby enhancing cell attachment, strengthening osteogenic responses, and potentially exerting antibacterial effects [23,24,25].

Emerging biologically inspired strategies, such as exosome-based coatings and cell membrane-functionalized surfaces, are attracting increasing attention. These approaches mimic natural intercellular communication mechanisms and actively modulate the peri-implant microenvironment by delivering bioactive molecules such as growth factors, cytokines, and microRNAs. As a result, they play a critical role in enhancing angiogenesis, osteogenesis, and immune regulation [26,27]. To address this, various biological modifications have been applied, including platelet-rich plasma (PRP)/platelet-rich fibrin (PRF), extracellular matrix (ECM), peptides (such as arginylglycylaspartic acid [RGD], P15 peptide, strontium-containing proteins, GL13K, and human beta-defensins), growth factors, signaling molecules, drugs, and antibacterial agents [9]. Specific peptides, such as P15, significantly accelerate osseointegration by stimulating osteoblast adhesion, cell migration, and early bone healing at the cellular level [28]. Coating titanium implant surfaces with bone morphogenetic proteins (BMPs) has been demonstrated to support bone regeneration [29]. In particular, BMP-2-coated titanium implants have shown increased surrounding bone density compared with acid-etched implants, as well as improved bone-to-implant contact and new bone formation compared with anodized implants [30,31]. In addition to these osteogenic effects, bactericidal peptides, such as GL13K, and sustained antimicrobial agents (e.g., tetracycline and vancomycin) actively inhibit biofilm colonization by exhibiting broad-spectrum antibacterial effects without impairing cell proliferation or tissue compatibility [32,33,34]. Although achieving the controlled release of these bioactive agents from implant surfaces remains a technical challenge, biological modifications represent a targeted and innovative strategy capable of accelerating osseointegration while simultaneously providing antibacterial protection, particularly in high-risk clinical scenarios. Consequently, modern biomimetic implant design increasingly relies on the integration of advanced surface engineering techniques with biologically inspired functionalization strategies. In this review, ‘biomimetic surface engineering’ is explicitly defined as strategies that intentionally replicate the hierarchical structures, biochemical compositions, or biological mechanisms of native tissues (e.g., bone extracellular matrix, mussel adhesion, or insect wing topographies) rather than merely providing passive bioactivity. While conventional surface modifications (such as standard acid-etching) aim to increase roughness, true biomimetic approaches actively guide cellular behavior and immunomodulation processes. Although previous reviews and book chapters have extensively discussed conventional implant coatings, this systematic review provides a novel perspective by strictly filtering the current literature (2021–2026) using this biomimetic definition. This review uniquely synthesizes how these nature-inspired functionalizations simultaneously address osseointegration, immune-instructive macrophage polarization, and antibacterial resistance—areas that have not been comprehensively updated in the recent literature.

The current literature indicates that physical, chemical, and biological surface modifications have the potential to accelerate osseointegration and reduce biofilm formation; however, most studies are based on heterogeneous experimental designs, different animal models, variable follow-up periods, and non-standardized outcome measures. In addition, comprehensive synthesis studies directly and systematically comparing biomimetic surfaces with conventional surfaces (e.g., machined and SLA) and holistically evaluating both biological markers (Alkaline Phosphatase (ALP) activity, osteogenic gene expression, and histomorphometry) and clinical parameters (BIC, Implant Stability Quotient (ISQ), and marginal bone loss) remain limited. This makes it difficult to determine the true effect size and clinical relevance of these modifications. This systematic review aimed to evaluate the effects of biomimetic surface modifications on dental implants on osseointegration and peri-implant bone regeneration compared with conventional implant surfaces based on current experimental and clinical evidence. Specifically, this study aims to determine the magnitude of these effects, their relationship with the underlying biological mechanisms, and the overall level of evidence. In this context, this review also seeks to identify the consistency of the current evidence base, methodological gaps, and priority areas for future research. Although not all engineering approaches identified in the broader implant literature fulfilled the strict biomimetic inclusion criteria of this review, these technologies constitute important platforms for enabling biomimetic functionalization and therefore represent a critical component of contemporary implant surface engineering.

## 2. Materials and Methods

### 2.1. Literature Search

This systematic review was conducted in accordance with the Preferred Reporting Items for Systematic Reviews and Meta-Analyses (PRISMA) guidelines (Table S1) [35], and the study protocol was prospectively registered in the International Prospective Register of Systematic Reviews (ID: 1348411). A literature search was conducted for studies published between 1 January 2021, and 31 January 2026, in the Web of Science, PubMed, and Scopus databases. The search strategy was created by combining relevant keywords using Boolean operators (AND, OR, NOT). The detailed search strategy used is summarized in Table 1. In the PubMed search, both MeSH terms and title/abstract keywords were used together. This approach aimed to increase the sensitivity of the search by including new publications that had not yet been fully indexed with MeSH terms. Additionally, manual hand-searching of relevant references and recently published articles was performed to ensure the inclusion of emerging biomimetic surface engineering strategies.

The search was restricted to the period between 2021 and 2026 to capture the most contemporary advancements in next-generation nanotechnology and biologically inspired coating. While several landmark investigations on basic bioactive modifications were published prior to this period, the last five years have marked a distinct shift towards multifunctional and immunomodulatory biomimetic interfaces.

### 2.2. Eligibility Criteria

This systematic review aimed to evaluate the effects of biomimetic surface modifications on dental implants for osseointegration and peri-implant bone regeneration compared with conventional implant surfaces. In this context, the research question was formulated as follows: Do biomimetic surface modifications enhance osseointegration and peri-implant bone regeneration in dental implants compared with conventional surfaces?

In this systematic review, the PICO framework was structured as follows: the population consisted of animal models and/or clinical patients receiving dental implants. The intervention included biomimetic surface modifications such as nano/micro topographical modifications, bioactive coatings (e.g., calcium phosphate [CaP] and graphene), and surfaces functionalized with peptides and growth factors. The comparison group comprised conventional implant surfaces (e.g., machined, SLA). The outcome measures included bone-to-implant contact (BIC%), implant stability quotient (ISQ), marginal bone loss (MBL), bone volume/total volume ratio (BV/TV), percentage of new bone formation, histomorphometric analysis findings, alkaline phosphatase (ALP) activity, and osteogenic gene expression levels (RUNX2, OCN, and OPN).

The inclusion criteria comprised original research articles published in English that evaluated dental implants with biomimetic surface modifications, reported data related to osseointegration or bone regeneration, clearly and comprehensively described their methodology, and included a statistical analysis.

The exclusion criteria were as follows: studies involving surface modifications without biomimetic characteristics; studies involving conventional surface treatments without a biologically inspired functionalization rationale or without biomimetic relevance; in vitro studies that did not involve dental implant applications; orthopedic implant studies unrelated to dental implants; studies that did not report data on osseointegration or bone regeneration; and studies that did not provide quantitative outcomes. Additionally, case reports, case series, narrative reviews, systematic reviews, meta-analyses, conference abstracts, and letters to the editor were excluded. Duplicate publications, methodologically inadequate studies, and articles for which the full text was not accessible were also excluded.

### 2.3. Data Extraction

Data extraction from the selected studies was performed independently by three reviewers (F.K., Z.D.G., and M.G.) using a predefined, standardized data collection form. For each study, the surface modification method (type of biomimetic approach, coating, and processing techniques), the micro/nanotopographical characteristics of the surface, the experimental design, and outcome variables were systematically recorded. The primary outcome parameters included bone-to-implant contact (BIC%), implant stability quotient (ISQ), marginal bone loss (MBL), bone volume/total volume ratio (BV/TV), percentage of new bone formation, histomorphometric analysis findings, alkaline phosphatase (ALP) activity, and osteogenic gene expression levels (RUNX2, OCN, and OPN). The main in vivo findings and key outcomes reported in each study were also documented. Inter-reviewer reliability for study selection and data extraction was evaluated using Cohen’s kappa statistic. A high level of agreement was achieved among the three reviewers (κ = 0.86), and any minor discrepancies were successfully resolved through discussion with the fourth reviewer (N.B.)

### 2.4. Synthesis of Results

This systematic review primarily focused on biomimetic implant modifications directly evaluated in experimental studies; however, complementary emerging technologies relevant to biomimetic functionalization were also narratively discussed to provide translational context. Owing to the substantial methodological and clinical heterogeneity among the included studies, such as fundamental differences in implant substrate materials (titanium, PEEK, and zirconia), diverse animal models, variable follow-up periods, and differing surface modification techniques, a quantitative meta-analysis could not be performed. Initial attempts to calculate the I^2^ statistic for primary outcomes, such as BIC and BV/TV, indicated high heterogeneity (I^2^ > 75%), which precluded meaningful pooling of data. Furthermore, the limited number of studies within distinct categories prevented robust subgroup analyses (e.g., comparing laser-textured titanium and coated PEEK). Consequently, a qualitative (narrative) synthesis was prospectively chosen as the most appropriate approach. Within this narrative framework, subgrouping by specific biomimetic strategies and implant materials was utilized to logically structure the analysis.

### 2.5. Risk-of-Bias Assessment

Since all studies included in this review were either in vivo animal experiments or in vitro material characterization studies, the methodological quality assessment was conducted based on the SYRCLE Risk of Bias Tool and the ARRIVE Guidelines, which are specifically designed to evaluate risk of bias in animal research. The assessment focused on three key indicators considered critical for methodological quality in animal studies by both approaches: (1) selection bias (randomization), (2) performance and detection bias, and (3) reporting of incomplete data or subject loss (attrition bias/dropouts). Each parameter was classified as low risk, unclear risk, or high risk.

## 3. Results

### 3.1. Study Selection

**Figure 1:**
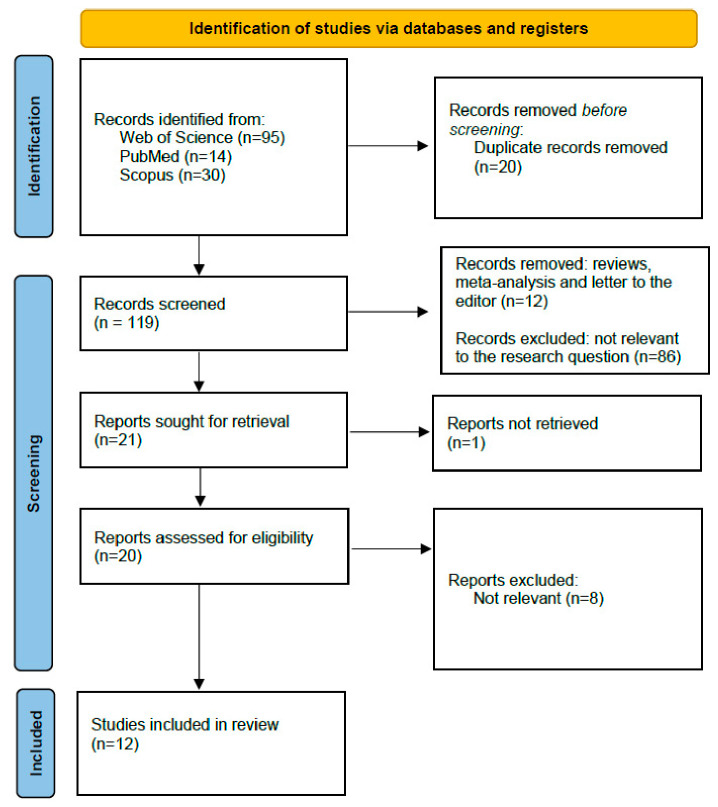
PRISMA 2020 flow diagram of study selection.

Following the literature search, a total of 139 records were identified from the electronic databases (Web of Science, n = 95; PubMed, n = 14; Scopus, n = 30). Prior to title and abstract screening, duplicate records (n = 20) and publications in the form of reviews, meta-analyses, and letters to the editor (n = 12) were excluded from the study. The remaining 107 records were screened at the title and abstract level; after excluding studies not relevant to the research question, 21 articles were selected for the full-text assessment. One study with an unavailable full text was excluded. Following the eligibility assessment, eight studies were excluded because they were not related to the research topic. Ultimately, 12 studies that met the predefined inclusion criteria were included in the systematic review (Figure 1).

### 3.2. Risk of Bias Results

**Figure 2:**
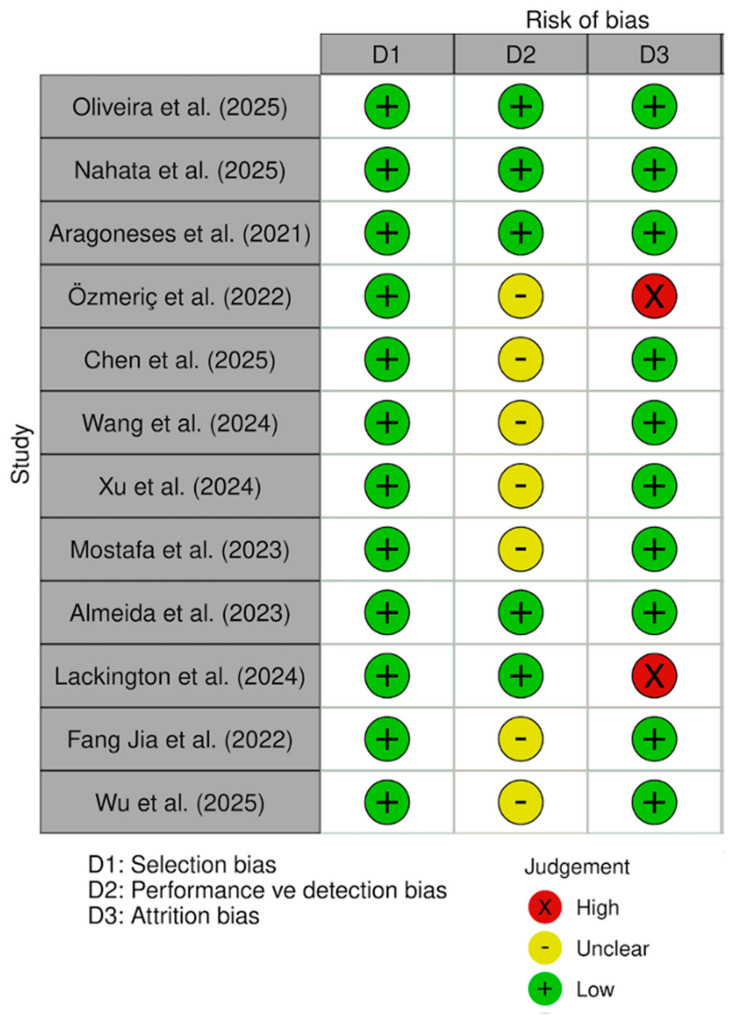
Risk of bias assessment for included studies using SYRCLE/ARRİVE domains [36,37,38,39,40,41,42,43,44,45,46,47].

The risk of bias assessment of the 12 selected in vivo animal studies, based on the SYRCLE and ARRIVE guidelines, is summarized in Figure 2. All studies reported random allocation of animals to experimental and control groups, indicating a low risk of selection bias. However, regarding performance and detection bias, seven studies lacked sufficient information on blinding and were therefore rated as unclear risk, while the remaining five studies employed methods such as double or triple blinding and were assessed as low risk. With respect to attrition bias (loss of subjects or implants), all studies except two [36,37] reported no losses during the follow-up period, indicating that the overall methodological quality of the included studies was generally high (Figure 2).

### 3.3. Classification of Biomimetic Surface Engineering Strategies

The studies included in this systematic review investigated biomimetic implant surface modifications designed to replicate specific biological principles observed in natural tissues and biological systems. To improve conceptual clarity and facilitate comparisons between approaches, the investigated strategies were categorized according to their underlying biomimetic mechanisms (Table 2).

#### 3.3.1. Modifications Mimicking Bone Structure

Approaches that mimic bone structure aim to reproduce the micro/nano-architecture of natural bone tissue through laser texturing, acid etching, and nanotopographic engineering. Femtosecond laser texturing and Nd:YAG laser processing have created trabecular-like architectures and nanoscale periodic structures that enhance fibrin deposition, osteoblast adhesion, and mechanical interlocking at the bone-implant interface [36,38]. In addition, specialized micromorphologies capable of directing cell movement have been obtained using advanced acid-etching formulations [39].

#### 3.3.2. Extracellular Matrix (ECM)-Inspired Biofunctionalization

Several studies have incorporated extracellular matrix-derived molecules and osteogenic signaling components, including fibronectin (FN) [40], bone morphogenetic protein-2 (BMP-2) [40], fibroblast growth factor-2 (FGF-2) [41], and platelet-rich fibrin (PRF) [38]. These bioactive coatings were designed to mimic the biochemical composition and signaling microenvironment of the native bone extracellular matrix, thereby promoting cell adhesion, proliferation, differentiation, and osseointegration.

#### 3.3.3. Mussel-Inspired Adhesive and Antibacterial Interfaces

In some studies, polydopamine (PDA) and DA-PAMAM-NH2 dendrimer systems, which provide stable surface adhesion in a wet oral environment, have been used [40,42]. These biomimetic coatings have demonstrated adhesion to implant surfaces by mimicking the catechol-mediated adhesion mechanism found in mussel adhesive proteins. Additionally, it has been reported that the mussel-inspired DA-PAMAM-NH2 dendrimer coating system provides strong antibacterial properties [42].

#### 3.3.4. Nature-Inspired Antibacterial and Immunomodulatory Structures

In one of the studies included in this review, zirconium phosphate (ZrP) nanonetwork surfaces inspired by insect wing (elytra) structures were developed. These biomimetic structures form highly porous frameworks that promote the polarization of macrophages toward the M2 phenotype while simultaneously enhancing bone integration [43].

#### 3.3.5. Functional Systems Mimicking the Periodontal Ligament

In one of the included studies, hydrogel-based coatings were developed to mimic the viscoelastic and biomechanical behavior of periodontal ligament tissue, with the aim of improving stress distribution and creating a more physiologically compatible implant interface [44].

#### 3.3.6. Osteogenic Ion and Mineral Mimicry

In some of the included studies, biomimetic calcium phosphate and strontium-doped hydroxyapatite coatings were used. It has been reported that these surface modifications enhance osteoconduction and promote bone regeneration around the implant by mimicking the mineral phase composition of natural bone tissue [45,46].

### 3.4. In Vitro Biological, Antibacterial, and Immunomodulatory Responses

The included studies evaluated the in vitro biological performance of biomimetic surface modifications in terms of cellular behavior, antibacterial activity, and immunomodulatory effects of the modified surfaces. Detailed data on these outcomes are presented in Table 3.

#### 3.4.1. Cell Adhesion and Proliferation

Titanium surfaces with high surface skewness (Ssk) and height (Sa) increase the number of adherent MG-63 cells by reducing their migration speed [39]. BMP-2/FN coatings on 3D-printed PEEK significantly enhanced cell spreading and viability compared to pure PEEK through a synergistic effect [40].

#### 3.4.2. Osteogenic and Angiogenic Differentiation

BMP-2 and Fibronectin (FN) modifications upregulated the expression of Runx2, ALP, and OCN genes in MC3T3-E1 cells and promoted vascularization [40]. In addition, PRF application on laser-induced nanotopographically modified polyetheretherketone (PEEK) surfaces markedly accelerated calcium phosphate (apatite) crystal formation [38].

#### 3.4.3. Immunomodulation

At the immunomodulatory and host-interaction levels, surface architectures actively dictate the immune response rather than acting as passive bystanders. For instance, elytra-inspired ZrP nanonetworks drive macrophage polarization exclusively towards the anti-inflammatory M2 phenotype, significantly downregulating pro-inflammatory markers and establishing a pro-healing microenvironment that accelerates mature bone formation [43].

#### 3.4.4. Antibacterial Activity

DA-PAMAM-NH2-coated titanium surfaces [42] and simvastatin/cefepime-loaded hydrogel-coated PEEK surfaces [44] effectively suppressed infection risk over a 4-week period, maintaining strong antibacterial activity against *S. aureus* and *E. coli* strains.

### 3.5. In Vivo Osseointegration Outcomes (BIC, BV/TV)

The included in vivo studies assessed the effects of biomimetic surface modifications on osseointegration and peri-implant bone regeneration using histomorphometric parameters, such as bone-to-implant contact (BIC) and bone volume fraction (BV/TV). The reported outcomes demonstrate the influence of different biomimetic strategies on peri-implant bone healing and integration. The detailed findings are presented in Table 4.

#### 3.5.1. Bone-to-Implant Contact (BIC)

In a sheep model with low-density bone, nanostructured hydroxyapatite (HAnano)-coated implants demonstrated a significantly higher BIC value at day 28 (82.27%) than acid-etched (DAA) surfaces [45]. PEEK implants modified with laser and PRF showed a substantial increase in BIC, rising from 56.43% to 84.80% [38]. Functionalization of titanium surfaces with FGF-2 increased the corrected BIC (BICc) from 43.08% to 60.48% [41], whereas ZrP nanonetworks formed on zirconia surfaces markedly enhanced BIC, reaching 52.24% [43].

#### 3.5.2. New Bone Formation and Bone Volume (BV/TV)

In rat mandibles, sulfonated PEKK implants exhibited greater new bone volume and superior osteogenic properties than titanium implants [47]. Drug-loaded hydrogel modifications applied to PEEK significantly increased the trabecular bone thickness [44]. Nanometer-thick boron nitride coatings have also been reported to promote greater new bone accumulation in the medullary cavity (15.70%) compared to control implants (8.23%) [37].

### 3.6. Biomechanical Stability and Compromised Conditions

The included studies also investigated the biomechanical stability of biomimetic implant surfaces and their performance under challenging conditions, including osteoporotic and low-density bone environments. The detailed findings are presented in Table 4.

#### 3.6.1. Removal Forces (Pull-Out and Reverse Torque)

TiZr implants textured with a femtosecond laser in rabbit tibias demonstrated greater mechanical interlocking in abiotic pullout tests than standard surfaces [36]. Similarly, the ZrP nanonetwork structure maximized the force required to detach the zirconia implants from the bone (pull-out force) [43].

#### 3.6.2. Occlusal Stress Distribution

Hydrogel coatings applied to PEEK surfaces absorb masticatory forces and uniformly distribute stress to the surrounding bone tissue [44].

#### 3.6.3. Osteoporosis Model and Low-Density Bone Model

In an ovariectomized rat model of osteoporosis, strontium-doped hydroxyapatite coatings exhibited significantly higher reverse torque values (4.32–4.4 N·cm) than control surfaces, ensuring stable fixation even in poor-quality bone [46]. In the low-density bone model of the sheep’s iliac crest, the HAnano surface exhibited significantly higher BIC (82.27%) and BAFo values on day 28 than the double-acid-etched (DAA) control group [45].

## 4. Discussion

This systematic review aimed to evaluate the effectiveness of biomimetic surface modifications developed to address key challenges limiting the clinical success of dental implants, including poor osseointegration, peri-implantitis, and stress-shielding. The findings from the 12 included in vivo and in vitro studies demonstrated that topographical, chemical, and biological modifications of titanium, zirconia, and PEEK/PEKK-based implant surfaces accelerated early stage osseointegration, enhanced fixation under conditions of low bone quality, and conferred antibacterial properties.

### 4.1. Topographical Modifications and Mechanical Regulation of Cellular Response

The micro- and nanostructures of implant surfaces are critical factors that directly influence osteoblast adhesion and migration. Jia et al. [39] demonstrated that surface skewness (Ssk) and mean height (Sa), created on titanium surfaces through advanced acid-etching formulations, can directly regulate the velocity and direction of osteoblast (MG-63) movement (cell migration velocity, CMV) without altering hemodynamic factors (e.g., blood perfusion). The observation that higher Ssk and Sa values significantly increased bone-to-implant contact (BIC) by enhancing cell adhesion highlights the mechanical influence of surface micromorphology on osseointegration.

Additionally, laser texturing has emerged as an alternative to the stochastic nature of conventional sandblasting/acid etching (SLA) methods. Lackington et al. [36] reported that trabecular-like microarchitectures and nanostructures produced by femtosecond lasers promote fibrin network formation, thereby enhancing early osseointegration through biological responses. Similarly, Mostafa et al. [38] demonstrated that nano-roughness generated by Nd:YAG laser on PEEK implants, when combined with platelet-rich fibrin (PRF), resulted in a high BIC value of 84.80%. This finding indicates that combining physical surface topography with biological agents (such as PRF) creates a synergistic bioactive effect.

### 4.2. Effects of Biochemical Coatings in Low-Density and Compromised Bone

In addition to topographical surface modifications, various bioactive coating strategies have been developed to further enhance the biological performance of dental implants. The mechanical strength of biometals and the bone-bonding potential of bioactive materials can be combined by applying bioactive coatings onto implant surfaces [48].

Such strategies are particularly important in clinical situations where achieving predictable and rapid osseointegration is challenging. Implant success in low-density bone regions, such as the posterior maxilla, or in osteoporotic patients, remains a significant clinical challenge. The studies included in this review confirm the critical role of calcium phosphate and hydroxyapatite (HA) derivatives in addressing these limitations. HA is a biologically stable type of calcium phosphate that mineralizes to strengthen the organic matrix without causing inflammation or immunogenicity [49].

It is composed of naturally occurring ions from physiological settings, and it has good osteoconductive and osteointegration properties. Several ion-substituted hybrid anchors paved the path for implant architecture featuring diverse biological activities. Ion-substituted HA coatings have been demonstrated to significantly enhance cell attachment, despite the possibility that they will negatively affect the growth and differentiation of cells attached to the coating surface. Moreover, the bioactivity and osteoconductivity of the titanium substrate can be enhanced by the HA layer [50,51].

Almeida et al. [45] demonstrated in a sheep model that nanostructured hydroxyapatite (HAnano) coatings resulted in significantly higher BIC (82.27%) and bone area fraction occupancy (BAFo) at 28 d compared with double acid-etched (DAA) surfaces. As a more advanced approach, Olivera et al. [46] showed that strontium-doped hydroxyapatite (HapSr) coatings significantly increased the reverse torque required to detach implants in an osteoporotic rat model compared with that of machined surfaces. The dual effect of strontium—stimulating osteoblastic activity while inhibiting osteoclastic resorption—makes it a strategic coating material for dental implantation in osteoporotic patients. As an alternative to the delamination drawbacks of conventional calcium phosphate coatings, Özmeriç et al. [37] investigated nanometer-thick boron nitride (BN) coatings, which were found to be biocompatible and showed a trend toward increased new bone formation in the medullary region; however, overall osseointegration and biomechanical performance were comparable to that of conventional titanium. Long-term mechanical stability also requires critical evaluation. Issues such as the risk of coating delamination during surgical insertion into dense cortical bone, degradation profiles of hydrogel networks, and coating durability under cyclic masticatory loading cannot be fully assessed within the standard 4-to-8-week follow-up periods typical of current in vivo models. Additionally, while outcomes in compromised models (e.g., osteoporotic rats or low-density bone) are promising, the limited total volume of such studies necessitates caution before extrapolating these findings to complex human clinical scenarios.

### 4.3. Alternative Materials to Titanium (PEEK/PEKK and Zirconia) and Surface Functionalization

While titanium’s high elastic modulus contributes to stress shielding, polymers such as polyether ether ketone (PEEK) and polyether ketone ketone (PEKK), with elastic properties closer to those of human bone, represent promising alternatives. However, their inherent bioinert nature limits their integration. The reviewed studies demonstrate that this limitation can be effectively overcome by chemical modification. Nahata et al. [47] reported that sulfonated PEKK (SPEKK) implants increased hydrophilicity and resulted in greater new bone volume in rat mandibles than titanium implants.

A more innovative strategy was presented by Xu et al. [44], who designed a hydrogel network (AA-AM) on 3D-printed PEEK to mimic the periodontal ligament (PDL). This structure functions as a “shock absorber” by distributing occlusal stress, simultaneously promoting osteogenesis through local simvastatin release, and providing antibacterial protection via cefepime release.

Wu et al. [43] developed a self-assembled zirconium phosphate (ZrP) nanonetwork inspired by insect wings (elytra) for zirconia (YSZ) implants. This highly porous structure promoted macrophage polarization toward the immunomodulatory anti-inflammatory M2 phenotype, significantly enhancing early osseointegration (as reflected by increased BIC) and improving the bonding strength with porcelain/resin materials.

A critical aspect of evaluating these biomimetic approaches is acknowledging the confounding effects of the underlying implant substrate. The biological responses observed in this study combined the results of studies utilizing titanium, zirconia, and PEEK/PEKK implants. Because the inherent surface energy, elastic modulus, and biological inertness of these substrates vary significantly, the success of a biomimetic coating on PEEK may not directly translate to titanium. Furthermore, the discussion of peri-implant bone regeneration must consider its long-term performance. Issues such as coating degradation, delamination of biomimetic layers under functional occlusal loading, and long-term chemical stability remain major challenges for surface-engineered implants and are currently underreported.

### 4.4. Long-Term Stability: Antibacterial and Osteogenic Synergy (Dual Action)

The prevention of peri-implantitis is as critical as osseointegration for the long-term survival of dental implants. Given that successful peri-implantitis management requires not only elimination of pathogenic biofilms but also restoration of peri-implant bone support, recent therapeutic strategies have increasingly focused on biomaterials capable of simultaneously controlling infection and supporting bone regeneration [52]. Recent surface-engineering approaches have therefore aimed to integrate antibacterial and osteogenic functions within a single coating system [53,54]. Such dual-action strategies are designed to promote re-osseointegration while simultaneously preventing bacterial recolonization, thereby enhancing the long-term clinical stability of dental implants [53,54].

Wang et al. [42] demonstrated that dopamine–PAMAM dendrimer conjugation (DA-PAMAM-NH2), inspired by mussel adhesive proteins, firmly adhered to titanium surfaces even in wet environments. This coating maintained strong antibacterial activity against *S. aureus* and *E. coli* after 4 weeks in simulated body fluid (SBF) and promoted osteoblastic mineralization, offering a durable solution for intraoral implant stability.

A similar dual-functional effect (biological functionality and mineralization) was reported by Chen et al. [40], who developed a biomimetic coating combining bone morphogenetic protein-2 (BMP-2) and fibronectin (FN) immobilized via polydopamine (PDA) on 3D-printed PEEK. This dual coating activated crosstalk between the Wnt/β-catenin and ERK/MAPK signaling pathways, enhancing angiogenesis in endothelial cells (HUVECs) and promoting significant new bone trabeculation in vivo.

As another example of direct growth factor application, Aragoneses et al. [41] covalently immobilized FGF-2 onto titanium surfaces using carboxyethylphosphonic acid, increasing mineralization rates in the cortical bone and raising corrected BIC values to approximately 60.48%.

In addition to BMP and FGF, coating materials incorporating other bioactive growth factors, including Transforming Growth Factor (TGF), Platelet-Derived Growth Factor (PDGF), and Vascular Endothelial Growth Factor (VEGF), have been extensively investigated [8,55]. These molecules have been reported to promote bone regeneration and enhance osseointegration by stimulating cellular proliferation, differentiation, and angiogenesis [56,57].

### 4.5. Limitations and Future Perspectives

Although the included studies provide strong in vivo evidence supporting the positive effects of biomimetic surface modifications on osseointegration, several important limitations should be considered. Methodologically, bounding the search strategy strictly to terms such as ‘biomimetic’ or ‘bio-inspired’ may have inadvertently excluded studies indexed under broader terms such as ‘osteoinductive’ or ‘biofunctionalized,’ although thorough hand-searching was employed to mitigate this. The inherent risk of publication bias must be acknowledged; preclinical studies demonstrating statistically significant positive enhancements are published more frequently, which may artificially inflate the perceived global efficacy of these novel surface modifications. Furthermore, the most common source of methodological uncertainty was insufficient reporting of blinding procedures. The predominance of preclinical evidence and the limited number of long-term randomized human clinical studies also hinder the direct translation of these findings into routine clinical practice.

Biomimetic surface modifications represent an innovative and multifunctional approach in implant dentistry that integrates physical, chemical, and biological functions at the implant surface. These strategies not only enhance osseointegration but also contribute to the active modulation of the peri-implant microenvironment by simultaneously regulating osteogenic, angiogenic, immunomodulatory, and antibacterial processes. Hybrid biomimetic approaches, in particular, appear to yield the most promising outcomes because of their ability to combine high biological activity with structural stability. Despite robust preclinical success, several critical translational challenges hinder immediate clinical adoption. Manufacturing complexity, high fabrication costs, and technical difficulties in sterilizing functionalized surfaces without denaturing bioactive proteins remain significant hurdles. Furthermore, regulatory approval pathways for hybrid ‘combination devices’ (integrating biomaterials with drugs or biologics) are exceptionally stringent. In addition, most studies employed long bone models (rat, rabbit, or sheep femur/tibia models), which do not fully replicate the unique anatomical structure of the mandible and maxilla, complexity of the oral microbiota, or dynamic biomechanics of masticatory forces [36,43,44,46]. Follow-up periods were generally limited to short- or medium-term durations (4–8 weeks), restricting the comprehensive evaluation of coating stability, surface degradation profiles, and long-term biological performance. Despite the strong preclinical evidence currently available, the lack of long-term human clinical studies remains a major limitation in this field. Therefore, future research should focus particularly on experimental models capable of simulating the oral environment over extended periods, as well as on large-scale human clinical studies.

This review, which evaluated the literature published between 2021 and 2026, provides strong evidence to support the in vivo success of biomimetic surfaces. However, more comprehensive systematic analyses are still needed to evaluate the long-term stability and antibacterial efficacy of advanced surface engineering techniques, such as anodization and electrophoretic deposition. Future well-designed clinical studies and standardized evaluation protocols will be critical for validating the reliability, safety, and clinical applicability of these advanced biomimetic surface modifications and establishing biomimetic implantology as a standard approach in routine clinical practice.

## 5. Conclusions

Biomimetic surface modifications enhance osseointegration via a series of interrelated biological mechanisms. Primarily, surface chemistry and nanotopographical features regulate protein adsorption, particularly adhesion molecules such as fibronectin and vitronectin, facilitating initial cell attachment. This process subsequently triggers intracellular signaling pathways, including integrin-mediated signaling, focal adhesion kinase (FAK), MAPK/ERK, and Wnt/β-catenin cascades, which collectively promote osteoblast proliferation and differentiation.

Furthermore, biomimetic surfaces modulate the host immune response by inducing macrophage polarization toward the anti-inflammatory M2 phenotype, thereby establishing a favorable microenvironment for tissue regeneration. Angiogenesis is enhanced by the incorporation or controlled release of growth factors, such as VEGF, leading to improved vascularization and nutrient delivery.

In addition, the incorporation of antibacterial coatings mitigates early bacterial colonization, thereby reducing the risk of peri-implantitis. Overall, the coordinated regulation of osteogenesis, angiogenesis, immune modulation, and antibacterial activity underscores the multifunctional potential of biomimetic surface engineering for improving implant integration.

Biomimetic approaches that integrate topographical laser engineering, sulfonation, mussel-inspired polymeric adhesives, and localized growth factor delivery represent significant advancements in dental implantology. These strategies transform implant surfaces from passive biocompatible materials into functional biointerfaces that actively guide tissue healing, regulate immune responses (e.g., M2 macrophage polarization), and provide resistance to infection. Nevertheless, well-designed advanced clinical studies are required to validate the long-term clinical efficacy and translational potential of these promising findings.

## Figures and Tables

**Table 1:** Database-specific search syntax.

**Web of Science**	TS = (“Dental Implants”) AND (“Surface Properties” OR “Surface Topograph” OR “Surface Modification” OR “Surface Treatment”) AND (“Biomimetic” OR “Bio-inspired” OR “Nature-inspired”) AND (“Osseointegration” OR “Peri-implant Bone Regeneration” OR “Bone Regeneration” OR “Bone-to-implant Contact”)
**PubMed**	((“Dental Implants”[Title/Abstract] OR “Dental Implant”[Title/Abstract]) AND (“Surface Properties”[Title/Abstract] OR “Surface Topography”[Title/Abstract] OR “Surface Modification”[Title/Abstract] OR “Surface Treatment”[Title/Abstract])) AND (“Biomimetic”[Title/Abstract] OR “Bio-inspired”[Title/Abstract] OR “Nature-inspired”[Title/Abstract]) AND (“Osseointegration”[Title/Abstract] OR “Peri-implant bone regeneration”[Title/Abstract] OR “Bone regeneration”[Title/Abstract] OR “Bone-to-implant contact”[Title/Abstract])
**Scopus**	TITLE-ABS-KEY (“dental implant”) AND TITLE-ABS-KEY (“surface properties” OR “surface topography” OR “surface modification” OR “surface treatment”) AND TITLE-ABS-KEY (biomimetic OR “bio-inspired” OR “nature-inspired”) AND TITLE-ABS-KEY (osseointegration OR “peri-implant bone regeneration” OR “bone regeneration” OR “bone-to-implant contact”)

**Table 2:** A comparative evaluation of biomimetic surface strategies in the studies included in the review.

Biomimetic Strategy	Biomimetic Principle	Representative Examples	Main Advantages	Main Limitations	Clinical Potential
Laser texturing	Bone hierarchical structure mimicry	Femtosecond laser, Nd:YAG laser	Enhanced osteoblast adhesion, fibrin retention, strong mechanical interlocking, improved BIC	High fabrication cost, equipment complexity	High
Sulfonation	Surface bioactivation and nano/microporous architecture formation	Sulfonated PEEK/PEKK	Increased hydrophilicity, improved osteogenesis, enhanced bone volume	Long-term chemical stability remains unclear	Moderate
Growth factor-functionalized coatings	Extracellular matrix (ECM) signaling mimicry	BMP-2, FGF-2, PRF, fibronectin	Strong osteogenic differentiation, angiogenesis, accelerated osseointegration	Burst release, short biological half-life, regulatory limitations	Moderate–High
Mussel-inspired coatings	Wet biological adhesion mimicry	Polydopamine, DA-PAMAM-NH2	Strong surface adhesion, antibacterial activity, bioactivity under wet conditions	Limited long-term clinical validation	High
Hydrogel-based systems	Periodontal ligament (PDL) mimicry	Drug-loaded AA-AM hydrogels	Stress distribution, biomechanical adaptation, controlled local drug delivery	Degradation control and long-term durability challenges	Experimental
Sr-doped hydroxyapatite coatings	Bone mineral phase mimicry	Strontium-containing Hap coatings	Improved fixation in osteoporotic bone, enhanced osteoconduction	Potential coating delamination and interface instability	High
ZrP nanonetworks	Immune-instructive and antibacterial architecture	Elytra-inspired zirconium phosphate structures	M2 macrophage polarization, enhanced osseointegration, improved bonding strength	Manufacturing complexity and limited translational evidence	Experimental

**Table 3:** In vitro biological, antibacterial, and immunomodulatory characteristics of included studies.

Authors	Implant Material	Surface Modification & Coating	In Vitro Tested Cell Types	In Vitro Outcome Variables & Biological Responses
**Lackington et al., 2024 [36]**	Titanium-zirconium	Femtosecond laser texturing (Nd:YAG)	Human Bone Progenitor Cells (HBPC)	Calcium quantification; Surface roughness (Sa, Sp, Sz). Enhanced fibrin retention.
**Özmeriç et al., 2022 [37]**	Titanium (SLA)	RF magnetron sputtering + Boron nitride (BN)	-	-
**Mostafa et al., 2023 [38]**	PEEK	Nd:YAG laser + UV or PRF coating	MG-63 Cells	SEM analysis; XRD analysis. Accelerated apatite crystal formation.
**Jia et al., 2022 [39]**	Grade IV Titanium	Chemical acid etching	MG-63 Cells	Cell migration velocity (CMV) and direction (CMD). Slowed migration rate promoted adhesion.
**Chen et al., 2025 [40]**	PEEK	PDA + BMP-2 + Fibronectin (FN)	MC3T3-E1 and HUVEC cells	Angiogenesis (CD31) and osteogenic genes. Synergistic osteogenic/angiogenic response.
**Aragoneses et al., 2021 [41]**	Grade IV Titanium	Carboxyethylphosphonic acid + FGF-2	MG63 osteoblast-like cells	Cell proliferation and viability.
**Wang et al., 2024 [42]**	Titanium Alloy (Ti6Al4V)	Mussel-inspired DA-PAMAM-NH2 dendrimer	MC3T3-E1 Cells	Inflammatory markers (TNF-α, BMP2); CFU. Maintained antibacterial activity under S. aureus infection.
**Wu et al., 2025 [43]**	Yttria-stabilized zirconia	Elytra-inspired ZrP nanonetwork	MC3T3-E1 and RAW 264.7 macrophages	Macrophage gene expression. Activated M2 anti-inflammatory macrophage polarization.
**Xu et al., 2024 [44]**	PEEK	Sulfonation + AA-AM hydrogel	MC3T3 and L929 cells	Cytocompatibility and local drug release dynamics.
**Almeida et al., 2023 [45]**	Titanium	Nanostructured hydroxyapatite (HAnano)	Osteoblasts	Cellular adhesion and morphology.
**Oliveira et al., 2025 [46]**	Titanium Alloy	Strontium-doped hydroxyapatite (HapSr)	-	-
**Nahata et al., 2025 [47]**	PEKK	Sulfonation with sulfuric acid	Human SCAP stem cells	Toxicity tests (Creatinine, SGOT, SGPT). Demonstrated high biocompatibility.

**Table 4:** In vivo osseointegration and biomechanical outcomes of included studies.

Authors	In Vivo Animal Model	Primary Surface Strategy	In Vivo Outcome Variables	Key Biomechanical & Histomorphometric Findings
**Lackington et al., 2024 [36]**	New Zealand rabbit tibia	Laser-induced trabecular microarchitecture	Pull-out Force (N)	Provided significantly higher abiotic pull-out strength compared with SLA surfaces.
**Özmeriç et al., 2022 [37]**	New Zealand rabbit tibia	Nano-BN coating	BIC (%); BATA; Removal torque	Higher new bone area in medullary cavity (15.70%) compared to controls.
**Mostafa et al., 2023 [38]**	Rabbit femur	Laser nanotopography + PRF	BIC (%)	Significantly increased BIC from 56.43% to 84.80%.
**Jia et al., 2022 [39]**	Beagle dogs (mandible)	Chemical acid etching	BIC; Intraosseous pressure; Micro-CT	Threefold increase in BIC ratio compared to control group during weeks 4–6.
**Chen et al., 2025 [40]**	Rat calvarial defect	PDA + BMP-2 + FN	BV/TV; Tb.Th; Tb.N; Histological scores	Achieved the statistically highest bone volume (BV/TV) values.
**Aragoneses et al., 2021 [41]**	Minipigs (Landrace)	FGF-2 Immobilization	BICc; BV/TV; Bone density	Increased corrected BIC (BICc) to 60.48% vs. 43.08% in controls.
**Wang et al., 2024 [42]**	Rat tibia (infection model)	DA-PAMAM-NH2 dendrimer	BMD; BV/TV; Trabecular thickness	Maintained high BMD and BV/TV values despite active S. aureus infection.
**Wu et al., 2025 [43]**	Japanese white rabbits	ZrP nanonetwork	BIC (%); Maximum pull-out force; Mature bone ratio	Reached 52.24% BIC and significantly increased pull-out strength vs. standard zirconia.
**Xu et al., 2024 [44]**	Rabbit femur	PDL-mimicking hydrogel	BV/TV; Bone density; Finite element stress	Distributed occlusal stress uniformly and enhanced new bone formation vs. pure PEEK.
**Almeida et al., 2023 [45]**	Sheep iliac crest (low-density bone)	Nanostructured hydroxyapatite (HAnano)	ITV; ISQ; BIC; BAFo	Achieved 82.27% BIC in low-density bone at day 28.
**Oliveira et al., 2025 [46]**	Osteoporotic female rats	Strontium-doped hydroxyapatite (HapSr)	Reverse torque; BIC; Linear bone contact	Provided significantly higher fixation (4.4 N·cm) in osteoporotic bone.
**Nahata et al., 2025 [47]**	Rat mandible	Sulfonated PEKK (SPEKK)	BV/TV; Trabecular thickness	Achieved greatest new bone volume compared with titanium and pure PEKK.

## Data Availability

Data are contained within the article or Supplementary Materials.

## Supplementary Materials

The following supporting information can be downloaded at: https://www.mdpi.com/article/10.3390/biomimetics11070460/s1, Table S1: PRISMA 2020 checklist [35]. This systematic review was registered in PROSPERO under the registration number CRD420261348411.
